# Multi-omics profiling of microbial ecology and non-volatile compounds across fermentation stages of spontaneous litchi (*Litchi chinensis* Sonn.) fermented vinegar-like beverage

**DOI:** 10.3389/fnut.2026.1908193

**Published:** 2026-07-30

**Authors:** Ting Wang, Honglian Liang, Yanglin Wu, Xiaomei Zhang, Shuqian Zhang, Zhengshan Wei, Wenshuo Li, Wenjin Song, Zhao Luo, Sam Al-Dalali

**Affiliations:** 1 School of Food and Health, Guilin Tourism University, Guilin, China; 2 Key Laboratory of Industrialized Processing and Safety of Guangxi Cuisine, (Guilin Tourism University), Education Department of Guangxi Zhuang Autonomous Region, Guilin, China; 3 School of Outdoor Sports, Guilin Tourism University, Guilin, China; 4 Department of Food Science and Technology, Faculty of Agriculture and Food Science, Ibb University, Ibb, Yemen

**Keywords:** litchi fruit, metagenomics, microbial ecology, spontaneous fermentation, vinegar-like beverage

## Abstract

**Introduction:**

Litchi fruit vinegar-like beverages (LVBs) are notable processed products derived from litchi fruit, yet few studies have focused on the systematic characterization of microbial and metabolic dynamics during their natural fermentation process.

**Methods:**

This work employed a comprehensive methodology integrating metagenomics and untargeted metabolomics based on UHPLC–MS/MS (Orbitrap Q Exactive HF-X) to elucidate the dynamic profiles of the microbial community and non-volatile metabolites, as well as their interrelations, across the various spontaneous fermentation stages of LVBs.

**Results:**

Metagenomic analysis indicated reduced microbial diversity and substantial structural changes within the community. Bacteria dominated the fermentation, accounting for 69.16 – 99.04% of the microbial community based on the taxonomically classified reads at the kingdom level. During the preliminary stage, *Leuconostoc*, *Enterobacter*, and *Klebsiella* were the prevalent genera. During the mid-fermentation stage, *Komagataeibacter* and *Lactiplantibacillus* emerged as the predominant genera in acid production. In the final stage, the microbial community was dominated primarily by *Zymomonas* and the *Acetobacteriaceae* family, including *Acetobacter* and *Komagataeibacter*. The non-targeted metabolomics study identified 2,382 metabolites through comprehensive database matching (in-house library, HMDB, KEGG, and metDNA algorithm) and stringent quality filtering (identification score > 0.5 and QC CV < 0.5), which were categorized into 20 distinct groups. Thirty seven metabolites, including amino acids, organic acids, and benzene derivatives, were identified as probable distinct differential metabolites based on a *p*-value threshold of *p* < 0.05, VIP > 1.0, and a fold change (FC ≥ 2 or ≤ 0.5) between consecutive fermentation stages in pairwise OPLS-DA of litchi vinegar-like beverage fermentation. Spearman correlation analysis revealed a highly organized ecological interaction network among dominant bacteria, physicochemical parameters, and non-volatile taste metabolites in the LVB fermentation system. *Zymomonas mobilis*, *Acetobacter pasteurianus*, *Leuconostoc suionicum*, and *Lactiplantibacillus plantarum* facilitated fermentation through metabolic synergy. Meanwhile, stage-specific enrichment of distinct Enterobacteriaceae species (*Enterobacter hormaechei*, and *Enterobacter quasiroggenkampii*) reflected species-level niche differentiation and resource competition, rather than a unified family-wide competitive behavior.

**Discussion:**

These findings provide a theoretical framework for engineering synthetic consortia and bioaugmentation approaches, informing the selection of starters and co-cultures to enhance LVB sensory and bioactive properties, alongside facilitating sfruit valorization.

## 1 Introduction

Litchi (*Litchi chinensis* Sonn.), a highly popular subtropical fruit, is widely consumed for its fleshy pulp (1, 2). It serves as both a food and a medicinal source. In traditional Chinese medicine (TCM), it is used to alleviate conditions such as indigestion, gastric ulcers, cough, and diarrhea (1). These effects are primarily attributed to its bioactive components, including carotenoids, vitamins, phenolics, flavonoids, proanthocyanidins, and anthocyanins (1, 3). Previous studies have demonstrated that its active constituents possess antioxidant, anti-inflammatory, anticancer, antidiabetic, antihyperlipidemic, antihyperglycemic, antihypertensive, antiviral, and immunomodulatory activities (4).

However, litchi has a limited harvest window and is particularly prone to postharvest browning and degradation, which considerably restricts storage stability and diminishes market value (5, 6). Litchi is frequently processed into pulp, juice, wine, and vinegar, thereby transforming highly perishable agricultural goods into more stable, value-added products and augmenting their bioactive constituents and related health benefits (7–9). Among these products, litchi vinegar typically uses commercial starter cultures in a two-step fermentation process: alcoholic fermentation is first carried out by *Saccharomyces cerevisiae*, followed by acetic fermentation with *Acetobacter pasteurianus*, resulting in a total acidity of over 50 g/L (10). Furthermore, co-fermentation with lactic acid bacteria (LAB) can enhance the vinegar’s softness, richness, and overall flavor, improve the liver’s total antioxidant capacity, and regulate lipid metabolism (11, 12). However, using commercial starter cultures leads to flavor homogenization and loss of raw-material typicality, which is detrimental to the development of regionally diversified products (13, 14).

In contrast, during spontaneous fermentation, indigenous microorganisms naturally present in raw materials, including yeasts, acetic acid bacteria, LAB, molds, and even bacteriophages, are key drivers of product quality (15). These microorganisms colonize the surfaces of fruits and other raw materials, grow rapidly, suppress pathogenic microbes, and, through their metabolism, produce flavor compounds and bioactive components such as organic acids, alcohols, and esters, thereby imparting unique sensory characteristics to the final product (16). This mechanism not only provides a theoretical basis for constructing synthetic microbial communities and applying innovative starter cultures in commercial fermentation but also lays the groundwork for developing complex flavor profiles (17). Notably, driven by social, economic, and environmental trends, the use of natural starter cultures in fermentation has become increasingly common, particularly in traditional, regional, and artisanal foods, especially those bearing Protected Designation of Origin (PDO) or Protected Geographical Indication (PGI) certification (18). Nevertheless, spontaneous fermentation is often unpredictable, making it difficult to ensure consistent product quality (19). Therefore, modern microbiological and multi-omics approaches are valuable for elucidating the underlying mechanisms, which may ultimately inform the rational design of starter cultures and fermentation strategies for improving the quality and functionality of spontaneously fermented products.

Not all spontaneous fermentation processes involving *Saccharomyces cerevisiae* and *Acetobacter pasteurianus* meet the legal acidity standard for vinegar. For example, (19) fermented pineapple peel waste spontaneously and obtained a beverage with a total acidity of only 3.03% (w/v, as acetic acid), which is below the U. S. FDA standard for acetic acid content in vinegar (≥4 g/100 mL). Because the acidity does not meet the legal requirement, such products cannot be labeled as “vinegar” and are consequently defined as “vinegar-like beverages” (19). Specifically, a vinegar-like beverage is an acid-containing fermented beverage produced from agricultural by-products such as fruit peels or fruit juices via spontaneous or controlled fermentation, with a total acidity (as acetic acid) lower than the legal standard for vinegar (19). These beverages typically retain organic acids, phenolic acids, and antioxidant activity generated during fermentation, thus exhibiting both the flavor characteristics of fermented beverages and certain health-promoting properties.

Research on litchi fruit vinegar-like beverages (LVB) remains limited. Elucidating microbial succession patterns and identifying key functional strains during LVB spontaneous fermentation would not only facilitate the rational selection of starter cultures and the optimization of fermentation performance but also lay the foundation for a stable production system. To achieve accurate process management, it is imperative to elucidate the temporal dynamics of microbial succession, as well as the functional roles and interaction mechanisms within the community.

Metagenomics has been a vital tool for elucidating the successional dynamics, functional capabilities, and interaction mechanisms of microbial communities during vinegar fermentation (20). In this study, we applied integrated metagenomic and untargeted metabolomic (UHPLC–MS/MS) approaches to systematically characterize the microbial succession and non-volatile metabolite dynamics during the spontaneous fermentation of litchi-derived vinegar-like beverages (LVBs). By correlating microbial taxa with physicochemical parameters and metabolite profiles, we aimed to establish a comprehensive microbe–metabolite association network underlying the flavor-relevant attributes of this fermented product. Rather than pursuing direct standardization, this study seeks to provide a theoretical basis for the rational design of starter cultures and targeted co-cultures in future fermentation practices.

## 2 Materials and methods

### 2.1 Chemicals

NaNO_2_ and Na_2_CO_3_ were obtained from Xilong Scientific Co., Ltd. (Guangdong, China). Al(NO_3_)_3_ was obtained from Sinopharm Chemical Reagent Co., Ltd. (Shanghai, China). NaOH was purchased from Aladdin Biochemical Technology Co., Ltd. (Shanghai, China). Folin–Ciocalteu phenol reagent, rutin, and gallic acid were purchased from Feijing Biotech Co., Ltd. (Fuzhou, China). Methanol, acetonitrile, and formic acid used in the mobile phase were of chromatographic grade (>99.9%) and were sourced from Merck (Darmstadt, Germany), Shanghai CINC High-Purity Solvents Co., Ltd. (Shanghai, China), and Aladdin Biochemical Technology Co., Ltd. (Shanghai, China), respectively. 2-Chloro-L-phenylalanine (purity ≥98%) was obtained from J&K Chemical (Beijing, China).

### 2.2 Preparation of LVB samples

Fresh lychees, free of pests and decay, were procured from a local commercial market (Qixing District, Guilin, Guangxi, China) for spontaneous vinegar-like beverage fermentation (LVBs). About 8 kg of fruit was placed in a 12 L lid-equipped tank and mixed with water to produce juice with a final sugar content of 15 Brix. The fermentation process was repeated three times in three separate tanks. Alcoholic fermentation was then conducted under anaerobic conditions using spontaneous bacteria from the fruit for 7 days. The mixture was then subjected to spontaneous acetic acid fermentation without filtration. To ensure homogeneity of the vinegar-like beverage, the fermentation tanks were stirred once every 2 days until fermentation ceased, and the fermentation continued for 49 days. Samples were collected on days 0, 7, 21, 35, and 49, representing the non-fermented stage (A), the alcoholic-fermented stage (B), the initial acetic acid fermentation stage (C), the mid acetic acid fermentation stage (D), and the late acetic acid fermentation stage (E). Three technical replicates were collected from each tank at each time point, yielding 45 samples in total (3 biological replicates × 3 technical replicates × 5 time points) across the entire fermentation period. All collected samples were placed into sterile 2 mL centrifuge tubes and stored at −80 °C until further analysis.

### 2.3 Determination of physicochemical indicators

The pH of the LVBs’ supernatant was measured using a calibrated pH meter (pH400, Alalis, Shanghai, China). Titratable acidity (TA) was determined by titrating the vinegar with 0.1 mol/L NaOH to an endpoint pH of 8.20 and expressed as acetic acid content in accordance with the Chinese National Standard GB/T 12456–2021.

### 2.4 Determination of flavonoids and polyphenols

The Folin–Ciocalteu method was used to determine total phenolic content (TPC) as described by Li et al. (21), with slight modifications. LVB samples were first diluted with purified water at a 1:10 ratio. A 20 μL aliquot of the diluted sample was mixed with 100 μL of Folin–Ciocalteu reagent (10%, *w/v*) and allowed to react for 3 min. Then, 80 μL of Na₂CO₃ solution (7.5%, *w/v*) was added, and the mixture was vortexed for 1 min. After incubation in the dark for 60 min, absorbance was measured at 765 nm using a microplate reader. The TPC concentration was calculated using a gallic acid equivalent (GAE) standard curve (Supplementary Figure S1A).

The total flavonoid content (TFC) was determined following Wu et al. (22), with appropriate adaptations. A 58 μL aliquot of the diluted sample was mixed with 16 μL of 5% (*w/v*) sodium nitrite solution, vortexed, and incubated at room temperature for 5 min. Subsequently, 16 μL of 10% (*w/v*) aluminum nitrate solution was added, followed by vortexing and incubation at room temperature for 6 min. Then, 160 μL of 4% (*w/v*) sodium hydroxide solution was added, and the mixture was vortexed and incubated at room temperature for 15 min. Absorbance was measured at 500 nm using a microplate reader. The TFC concentration was calculated using the rutin-equivalent standard curve (Supplementary Figure S1B).

### 2.5 Color determination

The color parameters, including L* (lightness), a* (redness), and b* (yellowness), were obtained using a spectrophotometer (CM-5, Konica Minolta, Tokyo, Japan).

### 2.6 DNA extraction and Illumina high-throughput sequencing

A 2 mL aliquot of the liquid sample was transferred to a sterile 2 mL microcentrifuge tube. The tube was centrifuged at 10,000 rpm for 3 min at room temperature. The supernatant was discarded, and the tube was inverted onto absorbent paper for 1 min to completely drain residual liquid. The resulting pellet was retained for DNA extraction. DNA was extracted from LVBs using the DNA Kit (Omega, M5635-02, United States) according to the manufacturer’s instructions. DNA concentration was measured with the Qubit™ dsDNA HS Assay Kit (Thermo Fisher Scientific, Q32851, United States), and DNA quality was assessed by 2% agarose gel electrophoresis (Thermo Fisher Scientific, United States) using an electrophoresis apparatus (FR-980A, Shanghai Furi Science & Technology Co., Ltd., China). DNA samples that met sequencing criteria were then sequenced with metagenomic sequencing on the NovaSeq 6,000 sequencers (Illumina, United States) provided by Sangon Biotech Co., Ltd. (Shanghai, China) (23). Quality filtering of raw reads was performed using Fastp, and removal of litchi host sequences was performed using Kneaddata (v0.12.0). Clean reads were taxonomically classified using Kraken2 (v2.1.2) against the NCBI NT database (confidence 0.2), and Bayesian re-estimated relative abundances at each taxonomic level (from domain to species) were done using Bracken (v2.5, default parameters).

The 42.650–44.450 million raw paired-end reads (150 bp) produced by each sample (A-D) were quality filtered and host-depleted to produce 6.27–6.50 Gb of clean data per sample. The raw read count of samples in group E ranged from 16.99 to 18.63 million, with 2.47 to 2.71 Gb of clean data per sample. The overall Q30 in all samples is above 95%, indicating good data quality. Sequencing statistics per individual sample is provided in Supplementary Table S1.

### 2.7 Analysis of non-volatile flavor compounds

Non-volatile flavor compounds in LVBs were analyzed by UHPLC–MS/MS on a Vanquish UHPLC system (Thermo Fisher, Massachusetts, United States) coupled to an Orbitrap Q Exactive HF–X mass spectrometer (Thermo Fisher, Massachusetts, United States) (24). For each fermentation stage, three technical replicates from each independent biological replicate (*n* = 3) were prepared. Samples were removed from −80 °C storage, thawed until no ice crystals remained, and vortexed for 10 s. Samples (100 μL) were transferred to pre-labeled 1.5 mL centrifuge tubes and resuspended in prechilled 80% methanol by vortexing. A 250 μg/mL internal standard of 2-chloro-L-phenylalanine (purity ≥98%) was prepared in 70% methanol and added to the mixture. The samples were incubated on ice for 5 min, then centrifuged at 15,000 × *g* for 20 min at 4 °C. A portion of the supernatant was diluted with LC–MS-grade water (Merck, Germany) to achieve a final concentration of 53% methanol. The mixture was vortexed for 20 min and centrifuged at 15,000 × *g* for 20 min at 4 °C. The supernatant was collected and filtered through a 0.22 μm microporous membrane prior to UHPLC–MS/MS analysis.

Chromatographic separation was performed on a Waters ACQUITY UPLC HSS T3 column (1.8 μm, 2.1 mm × 100 mm) maintained at 40 °C. The flow rate was set at 0.40 mL/min, and the injection volume was 4 μL. The mobile phases consisted of (A) 0.1% formic acid in ultrapure water and (B) 0.1% formic acid in acetonitrile. The gradient elution program was adjusted as follows: 0 min, 5% B; 2 min, 25% B; 4.5 min, 99% B; 4.5 min, 5% B; and 6 min, 5% B. The conditions used for mass spectrometry were as follows: Spray voltage: 3500 volts in positive ion mode; 3,200 volts in negative ion mode. Temperatures and gasses were controlled by the sheath gas flow rate of 30 Arbitrary Units (Arb); auxiliary gas flow rate of 5 Arb; ion transfer tube temperature of 320 °C; and vaporizer temperature of 300 °C. A full scan was obtained over the m/z range from 84 to 1,250 with a resolving power of 35,000, AGC target was 1.0 × 10^6. Data-dependent MS/MS scans were conducted at a resolving power of 17,500, with an AGC target of 2.0 × 10^5 and a signal threshold of 1.0 × 10^5. Collision energies were stepped at 30, 40, and 50 Electron-Volts (eV). The top *N* = 10 most intense precursor ions were fragmented with a dynamic exclusion duration of 3 s.

Raw UHPLC–MS/MS data were acquired as described by Hu et al. (25). Briefly, raw data were collected using SCIEX Analyst Workstation software (version 1.6.3). The raw mass spectrometry files were converted to mzML format using ProteoWizard, and subsequent feature extraction, alignment, and retention time correction were performed using the XCMS program. For data preprocessing, features with a missing rate exceeding 50% within each group were filtered out. Missing values were then imputed using a hybrid strategy: for features with a missing rate above 50%, one-fifth of the minimum value was used for imputation; for those with a missing rate below 50%, K-nearest neighbor (KNN) imputation was applied. To minimize systematic variation, peak areas were further corrected using the Support Vector Regression (SVR) method.

The filtered and corrected features were subjected to a multi-tiered library searching strategy using an in-house developed metabolomics analysis platform. Specifically, precursor ion (Q1) masses were initially matched against an in-house spectral library, followed by public databases (HMDB and KEGG), prediction libraries, and the metDNA algorithm—which expands identification coverage via metabolic reaction network propagation—in descending order of confidence priority. The precursor mass tolerance was set at 25 ppm for all library searches, with an additional retention time tolerance of 6 s exclusively applied to in-house library hits. For features with acquired MS/MS spectra, the observed fragmentation patterns were compared against reference spectra under a mass tolerance of 50 ppm. Annotation confidence was determined by a composite score integrating three weighted sub-scores: Fragment Score (weight = 0.1), Forward Search Score (weight = 0.3), and Reverse Search Score (weight = 0.6). Only candidates with a minimum MS/MS match score > 0.3 and a final composite score > 0.5 were retained as high-confidence identifications. For features lacking MS/MS data, tentative assignments were provided based solely on precursor mass matching (±25 ppm) without confirmation and were not included in the final high-confidence matrix.

To ensure analytical robustness, only metabolites with a coefficient of variation (CV) < 0.5 in quality control (QC) samples were retained. For features detected in both positive and negative ionization modes, the entry with the higher identification confidence and lower CV was selected for downstream analysis, yielding a final data matrix comprising 2,382 annotated metabolites.

To enable meaningful chemical grouping and comparative interpretation, these metabolites were further categorized using a two-tier hierarchical classification system. Classification annotations were integrated from three complementary sources: NPClassifier (for natural product-derived classes), ClassyFire/ChemOnt (for chemotaxonomic ontology), and the original database records (HMDB and KEGG). All conflicting or ambiguous assignments were manually curated and harmonized into a unified two-tier framework (Class I: broad superclass; Class II: specific subclass). This curated classification scheme subsequently served as the basis for all grouping strategies and comparative analyses.

We performed Principal coordinate analysis (PCoA) and orthogonal partial least squares discriminant analysis (OPLS-DA). To assess the model’s validity, OPLS-DA was evaluated using a permutation test. To screen for differential metabolites across stages, the criteria used were Student’s t-test *p*-value and variable importance in projection (VIP) of the first principal component of the OPLS-DA (*p* < 0.05 and VIP > 1). Inter-group differences were evaluated using one-way analysis of variance (ANOVA) followed by *post hoc* tests in SPSS 27.0.

### 2.8 Statistical analysis

Each sample was measured in three biological replicates (*n* = 3), with three technical replicates (*n* = 3). Data were presented as mean ± standard deviation (SD). Statistical analyses were performed using R. Significant differences in metabolites were assessed using the MetaboAnalystR package (*p* < 0.05). Unsupervised principal component analysis (PCA) and orthogonal partial least squares discriminant analysis (OPLS-DA) were performed to identify metabolite differences across fermentation stages (VIP > 1.0).

Alpha diversity indices were calculated for each sample using the vegan package to evaluate and compare microbial community richness and evenness. Beta diversity analysis was conducted using the Bray–Curtis dissimilarity index to construct a distance matrix among samples, followed by PCoA. Results were visualized using the Shengkeyun online platform^1^.

LEfSe analysis with the microeco package identified characteristic High-priority exploratory candidates of microbial succession across fermentation stages. The Kruskal–Wallis test (*p* < 0.05) was first used for initial screening, followed by Benjamini–Hochberg FDR correction for multiple comparisons. To further prioritize taxa with the strongest biological effects, an LDA score threshold of > 4.0 (well above the default 2.0) was applied. The results were visualized using ggplot2.

## 3 Results and discussion

### 3.1 Physicochemical indicators analysis

**Figure 1:**
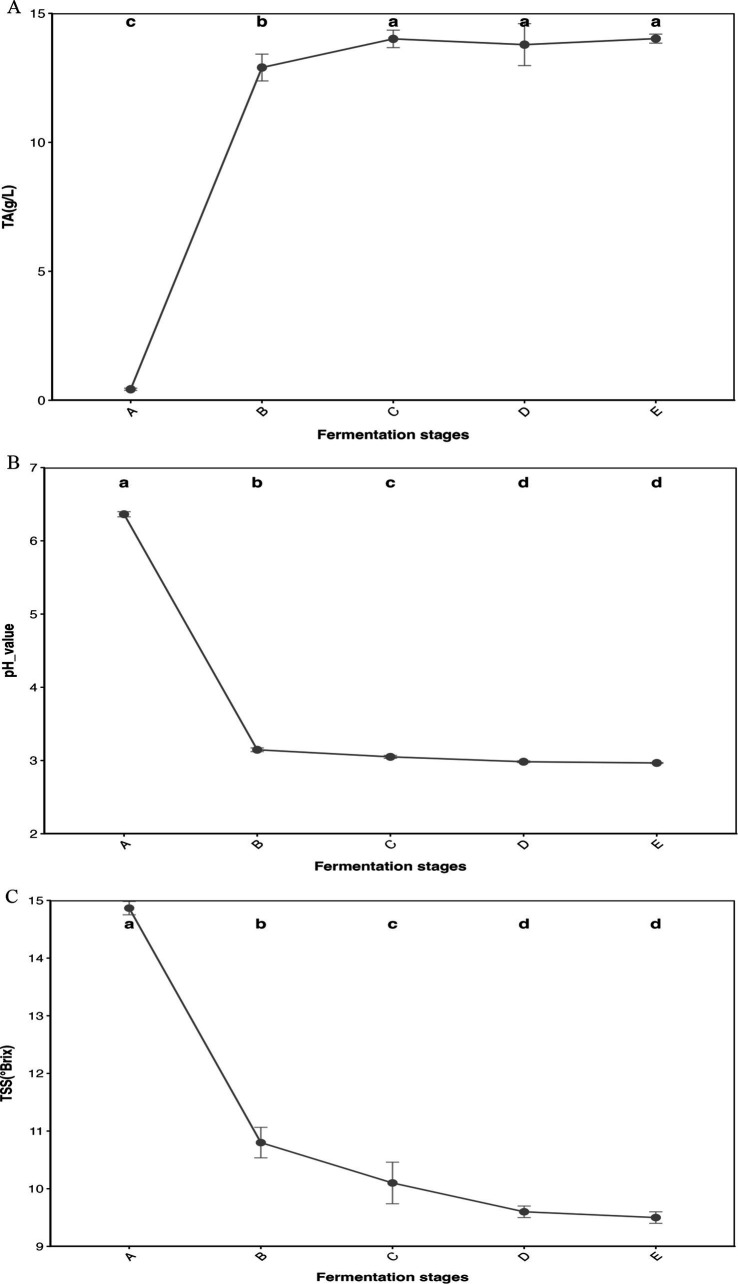
Changes of TA **(A)**, pH **(B)**, and TSS **(C)** during LVBs fermentation. A denoted the non-fermented stage, B denoted the alcoholic-fermentation stage, C denoted the initial acetic acid fermentation stage, D denoted the mid-acetic acid fermentation stage, and E denoted the late acetic acid fermentation stage. Different lowercase letters above each data point indicate significant differences (*P* < 0.05).

The physicochemical properties of LVB samples, including pH, TA, and TSS, which are environmental factors that influence microbial activity and metabolite production, were analyzed at different fermentation times. The results indicated that these basic indicators changed dynamically throughout the fermentation process (Figure 1). TA, a key indicator for monitoring acetic acid fermentation and product quality (26), increased sharply from 0.428 g/L to 12.90 g/L during the first 7 days (stage B) and reached a maximum of 14.020 g/L at the end of fermentation (stage E) (Figure 1A).

Meanwhile, pH and TSS decreased significantly during fermentation. pH dropped rapidly from 6.36 ± 0.03 to 3.14 ± 0.02 between stage A and stage B, then declined gradually to 2.96 ± 0.006 in stage E (Figure 1B). TSS also declined markedly from 14.87 °Bx to 9.5 °Bx. The decrease was pronounced from stage A to stage C, whereas no significant change was observed between stages D and E (Figure 1C). A similar trend was reported for vinegar by Yin et al. (26). This trend can primarily be explained by microbial consumption of carbohydrates in the fermentation system, a process that supports microbial maintenance while simultaneously generating large quantities of acids and flavor compounds.

### 3.2 Changes in TPC and TFC during fermentation of LVBs

Phenolic compounds are important bioactive constituents in vinegar (27), primarily derived from litchi raw materials (26), and they predominantly occur in bound forms (4). These bound phenolics are covalently linked to plant matrix components, such as pectin and polysaccharides, via ester bonds, making them resistant to hydrolysis (4). As fermentation progressed, the accumulation of acids and alcohol altered the environment, triggering the acid hydrolysis of bound phenolics. This process, together with microbial transformation, facilitated the release of free phenolics and modified the phenolic profile (28, 29).

As summarized in Table 1, LVB fermentation showed a series of development- and stage-specific changes in total polyphenols (TPC), total flavonoids (TFC), and colorimetric data across all five-time stages. The lowest TPC (0.399 ± 0.012 mg GAE/mL) and TFC (0.200 ± 0.010 mg RE/mL) values were observed in the non-fermented control (A), indicating the absence of bioactivity before the initiation of microbial-induced hydrolysis. The significant increase in TPC to 0.569 mg GAE/mL suggested that alcoholic fermentation (B) promoted the release of bound phenolics from the fruit matrix via yeast-mediated hydrolysis (30, 31). The peak TPC and TFC were observed at the early and intermediate stages of acetic acid fermentation (stages C and D, respectively); interestingly, the highest TFC value of 0.377 ± 0.014 mg RE/mL was observed in stage D, indicating that acetic acid bacteria promoted enhanced enzymatic deglycosylation of glycosylated flavonoids. This trend is consistent with other studies reporting improved polyphenol bioavailability from two-stage fermentation that involves enzymatic deglycosylation and cell wall destruction. While the TPC reached a maximum of 0.636 ± 0.019 mg GAE/mL on day 21, and TFC exhibited a similar trend, peaking at 0.377 ± 0.014 mg RE/mL on day 35, followed by a decline. This decreasing trend may be attributed to the oxidation or degradation of phenolic compounds, facilitated by oxygen exposure and low pH during the acetic acid fermentation stage (26). Nevertheless, the fermented levels of TPC and TFC remained higher than their initial values.

**Table 1:** Changes of TPC, TFC, and color during the fermentation of LVBs.

Group	TPC (mg GAE/mL)	TFC (mg RE/mL)	L* value	a* value	b* value
A	0.399 ± 0.012^c^	0.200 ± 0.010^e^	42.287 ± 0.246^ab^	−2.727 ± 0.074^c^	0.287 ± 0.548^c^
B	0.569 ± 0.020^b^	0.234 ± 0.007^d^	41.037 ± 0.015^c^	14.643 ± 0.372^a^	13.543 ± 0.071^b^
C	0.636 ± 0.019^a^	0.337 ± 0.033^b^	42.573 ± 0.374^a^	10.563 ± 0.049^b^	16.177 ± 0.151^a^
D	0.611 ± 0.009^a^	0.377 ± 0.014^a^	41.990 ± 0.078^b^	10.940 ± 0.173^b^	16.280 ± 0.026^a^
E	0.600 ± 0.030^ab^	0.280 ± 0.011^c^	40.527 ± 0.270^d^	10.020 ± 1.058^b^	16.630 ± 0.080^a^

A denoted the non-fermented stage, B denoted the alcoholic-fermentation stage, C denoted the initial acetic acid fermentation stage, D denoted the mid-acetic acid fermentation stage, and E denoted the late acetic acid fermentation stage. Different lowercase superscript letters in the same column indicate significant differences (*P* < 0.05).

### 3.3 Changes in color of LVBs during fermentation

Color is an important parameter for evaluating the quality of fruit vinegar-like beverage products (32). As shown in Table 1, significant differences (*p* < 0.05) were observed in the color parameters (L*, a*, and b* values) of LVBs across fermentation stages. The L* value, associated with sample lightness, changed significantly throughout fermentation (*p* < 0.05). The dramatic change in a* values from significantly negative (−2.727 ± 0.074) in stage A to strongly positive in stages B-E represents a color shift from green to red-orange, due to oxidative degradation of polyphenols and pigment conversion. The a* value increased initially, peaked at 14.643 ± 0.372 in stage B, and then decreased, with no further significant change after stage C. This trend may be attributed to the release of phenolic compounds, such as anthocyanins, from the litchi pericarp during fermentation, which markedly increased the redness (a* value) of the vinegar and vinegar-like beverage (33). However, anthocyanins are pH-sensitive, and their degradation under acidic conditions during the late stage of fermentation reduced redness (34). This decreasing trend in the a* value was consistent with the observed decrease in TFC and increase in the b* value from stage C to stage E in this study.

The b* value followed a trend similar to a*, increasing continuously to a peak of 16.630 ± 0.080 in stage E, indicating enhanced yellowness. This may be related to browning during vinegar fermentation; brown or black pigments generated by the Maillard reaction or caramelization can influence the b* value (33). Additionally, Verzelloni et al. reported that under low-pH conditions, flavonoids may undergo condensation reactions, forming yellow polymers that contribute to the development of vinegar yellowness (35). These changes are consistent with the observed decrease in TFC and increase in b* value from stage D to stage E in our study. In brief, fermentation significantly altered the color of LVBs, and the vinegar’s brightness decreased from white to reddish-yellow.

### 3.4 Metagenomic data overview

Metagenomic sequencing was employed to investigate the microbial composition, successional dynamics, and functional characteristics during the spontaneous fermentation of LVBs. Supplementary Table S1 summarizes the main quality metrics for sequencing and gene predictions for all means of 15 LVB samples (A1-A3, B1-B3, C1-C3, D1-D3, E1-E3) at the five fermentation stages, with three replicates each. The raw read counts were high for almost all sample types (averaging 42–44 million), but the stage E samples produced significantly fewer reads (around 17–19 million). This may indicate reduced microbial biomass or changes in community composition by the time of late-stage acetic acid production. Although these samples had less sequence information after removing poor-quality sequences, they still had an impressive amount of high-quality sequence data (~6.0–~6.5 GB) compared with samples from earlier fermentation stages. Also, the percentages of base calls with Quality Scores of 20 and 30 were very high (>99 and >95%, respectively) across nearly all samples analyzed. These figures confirm excellent sequencing quality, which will be useful for subsequent metagenome analysis. In addition, GC percentages of the cleaned DNA across all sample types were similar (around 48–52%). *De novo* assembly was performed using MEGAHIT (version 1.2.9), for which the number of assembled contigs ranged from approximately 35,000 to ~77,000; their average N50 lengths averaged about 1,600 to ~2,400 bp; and their average N90 lengths averaged about 580 to ~625 bp. These values represent acceptable levels of assembly contiguity across all of the fermentation stages. Samples in the stage A category appeared to produce the highest numbers of contigs and the longest N50, possibly indicative of increased diversity prior to community selection via fermentation processes. Conversely, samples in stage E produced significantly fewer contigs and shorter assemblies, consistent with the much lower total sequence data generated at this stage. As expected, gene prediction yields mirrored the trends described above. Predicted gene numbers ranged from approximately 70,000 to ~135,000. Average gene sizes were virtually identical for all categories (approximately 595–630 bp), and %GC’s for predicted genes fell in a similar range (around 52–57%) using Prodiga software. The overall stability and accuracy of open reading frame (ORF) prediction are supported by both sets of gene-size parameters. Reduced gene predictions in stage E samples are attributable to lower sequencing depth rather than a biological reduction in the gene repertoire. Overall, the sequencing and assembly metrics shown in Supplementary Table S1 provide evidence that the quality of most of the sequence data is sufficient to enable meaningful comparison of microbial community dynamics over the course of LVB fermentation.

### 3.5 Dynamics of microbial communities during fermentation of LVBs

The diversity of the microbial community is a critical determinant of the fermentation system’s functional stability and ultimately dictates the distinct flavor profile. Alpha and beta diversity analyses were used to elucidate microbial diversity and structural dynamics across different stages of spontaneous fermentation in LVBs.

**Figure 2:**
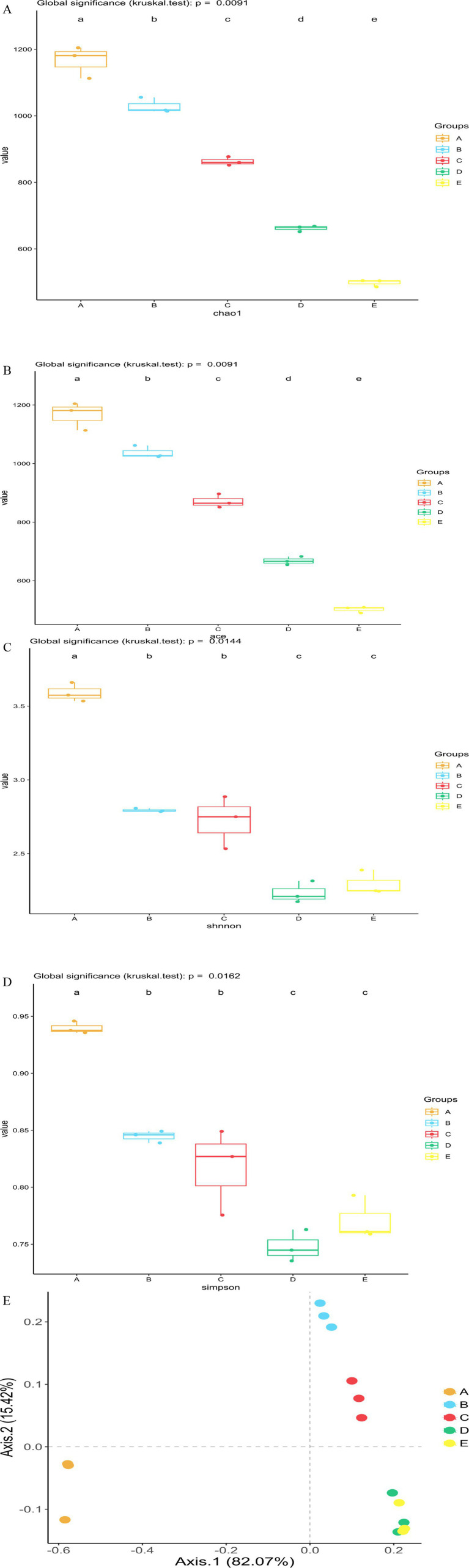
Diversity of microbial communities during LVBs fermentation. Alpha diversity indices (Chao1, ACE, Shannon, Simpson) at the species level in different fermentation stages of LVBs **(A–D)**, respectively. Principal coordinates analysis **(E)** based on Bray-Curtis distances of beta diversity at the genus level of microbial communities at different fermentation stages of LVBs. A denoted the non-fermented stage, B denoted the alcoholic-fermentation stage, C denoted the initial acetic acid fermentation stage, D denoted the mid-acetic acid fermentation stage, and E denoted the late acetic acid fermentation stage. Different lowercase letters above each data point indicate significant differences (*P* < 0.05).

α-diversity was assessed using four indices (Figures 2A–D): ACE, Chao1, Shannon, and Simpson, which capture temporal and spatial diversity and richness of microorganisms. Higher ACE and Chao1 indices indicated greater richness, while higher Shannon and Simpson indices collectively indicated greater microbial community diversity. In stage A, the sample exhibited the highest values of the ACE, Chao1, and Shannon indices, reflecting the greatest species richness and diversity. As fermentation progressed, these indices declined markedly, with stage E showing the lowest richness. These results suggest a gradual reduction in microbial diversity during fermentation, which may be attributable to increasing environmental stress and selective pressure. The continual rise in TA content and the concomitant decrease in LVBs’ pH inhibited the growth of certain bacterial populations, thereby driving the observed decline in overall community diversity.

Beta diversity was analyzed to assess structural differences in microbial communities across stages. PCoA based on Bray–Curtis distances (Figure 2E) showed clear separation of samples along the first axis, which explained 82.07% of the total variation. Excluding stages D and E, which clustered closely, the remaining stages were distinctly separated in the PCoA plot, suggesting microbial compositional similarity between D and E.

### 3.6 Microbial succession in LVBs during fermentation

#### 3.6.1 Microbial composition analysis

Microbial taxonomic annotation of LVBs revealed 23 phyla, 631 genera, and 2,164 species using the NT database (Supplementary Table S2). At the phylum level, 17 bacterial phyla (*Pseudomonadota*, *Actinomycetota*, *Bacillota*, *Bacteroidota*, *Myxococcota*, *Deinococota*, *Planctomycetota*, *Acidobacteriota*, *Bdellovibrionota*, *Verrucomicrobiota*, *Thermodesulfobacteriota*, *Deferribacterota*, *Cyanobacteriota*, *Chloroflexota*, *Candidatus Saccharimonadota*, *Campylobacterota*, and *Abditibacteriota*) and 3 fungal phyla (*Ascomycota*, *Basidiomycota*, and *Oomycota*) were detected in all samples.

At the genus level, the profile comprised 441 bacterial genera (representing 1861 species) and 123 fungal genera (representing 303 species) (Supplementary Table S2). The microbial community composition shifted markedly during fermentation, with bacterial abundance increasing from 69.16 to 99.04% as fungal abundance decreased from 11.50 to 0.06% (Supplementary Figure S2). This pattern indicates a clear trend in microbial community succession and underscores the critical role of bacteria in the dynamic process of spontaneous fermentation in LVBs. This observed succession pattern is similar to the finding of Zhu et al., namely the presence of shared critical microbial taxa during the fermentation of distinct vinegars (36).

**Figure 3:**
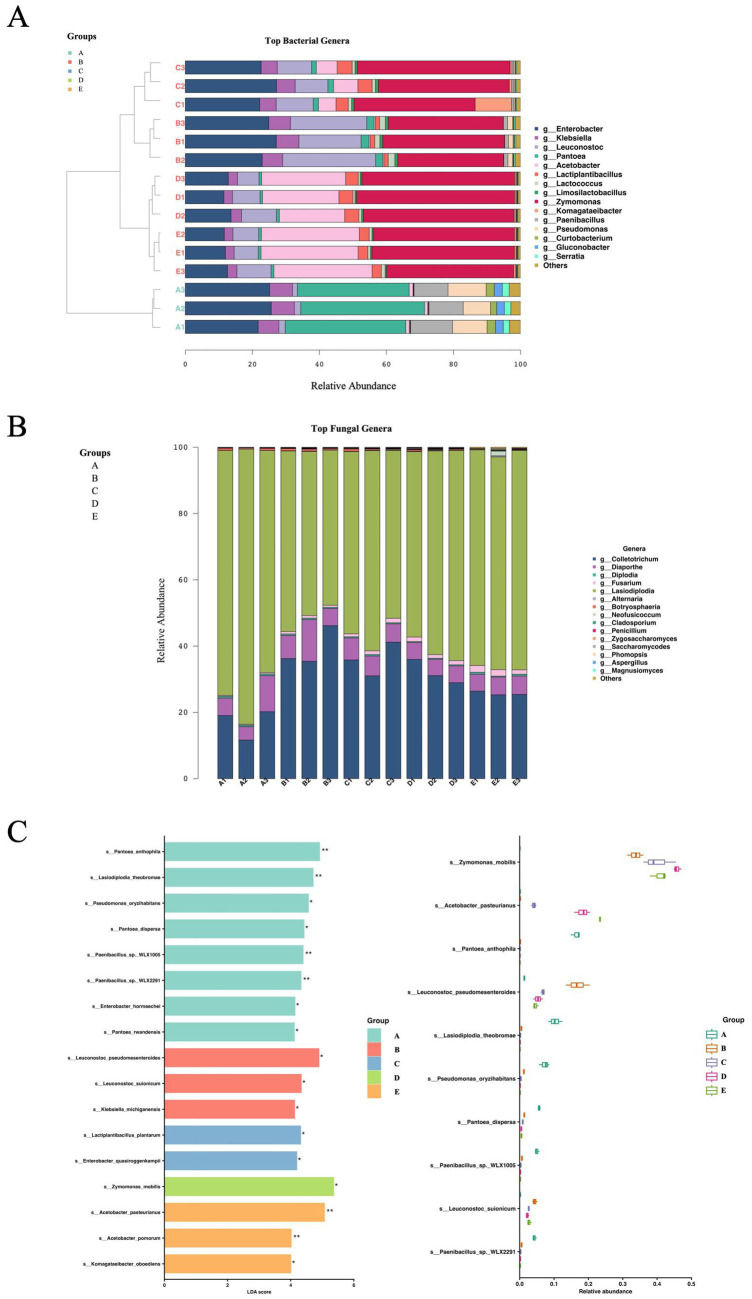
The microbial community temporal succession during LVB’s spontaneous fermentation. **(A)** Relative abundance of the bacterial community at the genus level. **(B)** Relative abundance of the fungal community at the genus level. **(C)** Significant microbial biomarkers identified by LEfSe. A denoted the non-fermented stage, B denoted the alcoholic-fermentation stage, C denoted the initial acetic acid fermentation stage, D denoted the mid-acetic acid fermentation stage, and E denoted the late acetic acid fermentation stage. Different lowercase letters above each data point indicate significant differences (*P* < 0.05).

The succession of bacterial community structure in LVBs across fermentation stages is shown in Figure 3A. At the genus level, the community in stage A was primarily composed of *Pantoea* (35.43%), *Enterobacter* (24.13%), *Paenibacillus* (10.93%), and *Pseudomonas* (9.94%). In stages B and C, *Zymomonas* (34.10 and 40.26%), *Enterobacter* (24.99 and 24.00%), and *Leuconostoc* (16.67 and 10.36%) were the dominant species. In stages D and E, *Zymomonas* (45.86 and 40.84%), *Acetobacter* (22.44 and 29.16%), and *Enterobacter* (12.64 and 12.04%) were the dominant species. Specifically, the relative abundances of *Lactococcus*, *Limosilactobacillus*, and *Leuconostoc* in LVBs increased and then decreased during fermentation, reaching peak values of 1.74, 0.84, and 23.00%, respectively, in stage B. The abundance of *Lactiplantibacillus* reached its maximum of 4.13% in stage C. In addition, the relative abundance of *Acetobacter* increased steadily from stage B to E (during acetic acid fermentation). In contrast, the relative abundances of six genera, including *Pantoea*, *Paenibacillus*, *Pseudomonas*, *Curtobacterium*, *Gluconobacter*, and *Serratia*, declined consistently over the entire fermentation period. Separately, the abundance of *Klebsiella* decreased during acetic acid fermentation, while that of Enterobacter declined after day 21 (in stages D and E).

A dynamic succession of the fungal community was observed at different stages of LVB’s spontaneous fermentation, as shown in Figure 3B. At the genus level, the dominant taxa during LVB’s fermentation were *Colletotrichum* and *Lasiodiplodia*. The relative abundance of *Colletotrichum* increased initially, then decreased on day 7 (stage B), fluctuating between 16.96 and 39.27%. In contrast, *Lasiodiplodia* showed an inverse pattern, with abundance ranging from 50.30 to 74.69%. Notably, the relative abundances of *Saccharomycodes* and *Fusarium* increased significantly, reaching 0.17 and 1.78%, respectively, in stage E. Conversely, the abundance of *Botryosphaeria* decreased continuously throughout fermentation.

#### 3.6.2 High-priority exploratory candidates of microbial succession

Microbial biomarkers at different stages of LVB fermentation were identified using the Linear Discriminant Analysis Effect Size (LEfSe) algorithm (37). A total of 2,164 species were subjected to the Kruskal–Wallis test, yielding 261 genera and 778 significant species (FDR < 0.05) (Supplementary Table S3), indicating that the fermentation process dramatically reshaped the microbial community structure. Data were visualized using linear discriminant analysis (LDA) with a score threshold > 4 (Figure 3C). The number of significant genera identified at stages A-E was 5, 3, 2, 1, and 2, respectively. Specifically, *Pantoea*, *Lasiodiplodia*, *Pseudomonas*, *Paenibacillus*, and *Enterobacter* were significantly enriched in the raw materials at stage A.

Lactic acid bacteria are common in traditional fermented foods (38). In stage B, *Leuconostoc pseudomesenteroides* (4.90), *Leuconostoc suionicum* (4.34), and *Klebsiella michiganensis* (4.12) were significantly enriched (Figure 3C). *Leuconostoc* was the primary initiator and dominant microbiota at the early stage of litchi vinegar-like beverage fermentation, consistent with previous reports (39–41). This finding indicates that *Leuconostoc* exhibits strong adaptability to the litchi substrate, enabling rapid colonization of the ecological niche and initiation of fermentation (40). Additionally, *Leuconostoc* is a heterotypic lactic acid fermenter that can utilize monosaccharides and disaccharides to produce glucose, lactic acid, acetic acid, and ethanol, and to generate carbon dioxide during fermentation, thereby lowering the pH of the fermentation environment and inhibiting opportunistic pathogens such as *Enterobacteriaceae* (41). *Leuconostoc’s* ability to inhibit *Enterobacteriaceae* is insufficient, and acid- and salt-tolerant *Enterobacteriaceae* are more likely to overcome the ecological barrier and occupy the niche (42). In stage C, *Lactiplantibacillus plantarum* and *Enterobacter quasiroggenkampii* had become the dominant species (Figure 3C), indicating that *Lactiplantibacillus plantarum*, with high acid resistance, gradually replaces *Leuconostoc*, which has poor acid resistance and is microaerophilic, and further produces acid to reduce the pH of the system (40). This succession reflects the adaptive shift of microbial communities from acid-sensitive to acid-tolerant populations in response to the acidified ecological niche during vinegar fermentation (43). Additionally, Hu et al. (44) found that *Lactiplantibacillus plantarum* plays a crucial role in the formation of flavor compounds by increasing the levels of lactic acid and aromatic esters in vinegar.

In stage D, *Zymomonas mobilis* (5.36) was dominant. In contrast, *Acetobacter pasteurianus* (5.07), *Komagataeibacter oboediens* (4.03), and *Acetobacter pomorum* (4.01) were dominant in stage E (Figure 3C). These findings indicated that *Zymomonas*, *Acetobacter*, and *Komagataeibacter* were the dominant microbiota during acetic acid fermentation in LVBs. This succession pattern resembles that of traditional cereal vinegar but also shows distinct differences (36). In cereal vinegar fermentation, acetic acid bacteria (AAB), including *Acetobacter* and *Komagataeibacter*, are the key functional group responsible for oxidizing ethanol to acetic acid during the acetic acid fermentation stage (45). Ethanol typically originates from exogenous addition or is produced from glucose by *Saccharomyces cerevisiae* (45, 46). In the present system, *Saccharomycodes ludwigii* and *Z. mobilis* were the primary taxa with known ethanologenic potential, suggesting that they may serve as the main ethanol suppliers for subsequent AAB activity. Nevertheless, as metagenomic data reflect genetic potential rather than real-time metabolic activity, the actual ethanol-producing contribution of *Z. mobilis* during this stage requires validation through functional approaches such as metatranscriptomics or metabolic flux analysis.

*Zymomonas mobilis* is a Gram-negative, facultatively anaerobic bacterium that metabolizes sucrose, glucose, and fructose via the Entner–Doudoroff (ED) pathway and converts them to ethanol using the key enzymes pyruvate decarboxylase (Pdc) and alcohol dehydrogenase (Adhs) (47, 48). Compared to *S. ludwigii*, *Z. mobilis* exhibited higher efficiency in alcohol production (49). Thus, this fermentation process does not require strict oxygen control, which not only simplifies operations but also reduces equipment investment and overall production costs. However, because it lacks amylase and maltase, *Z. mobilis* is suitable for fuel ethanol production from starch-based substrates after a saccharification step (47). Currently, *Z. mobilis* is primarily used to produce alcoholic or low-sugar beverages such as pulque, palm wine, and taberna (49). In contrast, its application in cereal-based fermented foods requires the addition of starch-hydrolyzing enzymes or co-cultivation with amylolytic strains (48).

Additionally, the sustained high acidity during vinegar fermentation likely suppressed acid-sensitive taxa, including members of the family *Enterobacteriaceae* (*E. hormaechei*, and *E. quasiroggenkampii*, *and Klebsiella michiganensis*) and the family *Erwiniaceae* (*P. anthophila* and *P. rwandensis*). Importantly, the stage-enriched taxa identified through LEfSe (e.g., *Zymomonas*, *Acetobacter*, *Leuconostoc*, and *Lactiplantibacillus*) exhibited consistent successional patterns and are supported by known functional roles in fermentation. However, given the limited sample size (*n* = 3 per group) and the exploratory nature of this untargeted metagenomic survey, these taxa should be considered as high-priority candidates rather than definitive biomarkers. Targeted validation via qPCR, cultivation, or absolute quantification in future independent cohorts is warranted. Nonetheless, our findings collectively demonstrate a distinct temporal succession of the microbial community during the spontaneous fermentation of LVBs.

### 3.7 Metabolite analysis during fermentation

#### 3.7.1 Multivariate statistical analysis of LVB metabolites

**Figure 4:**
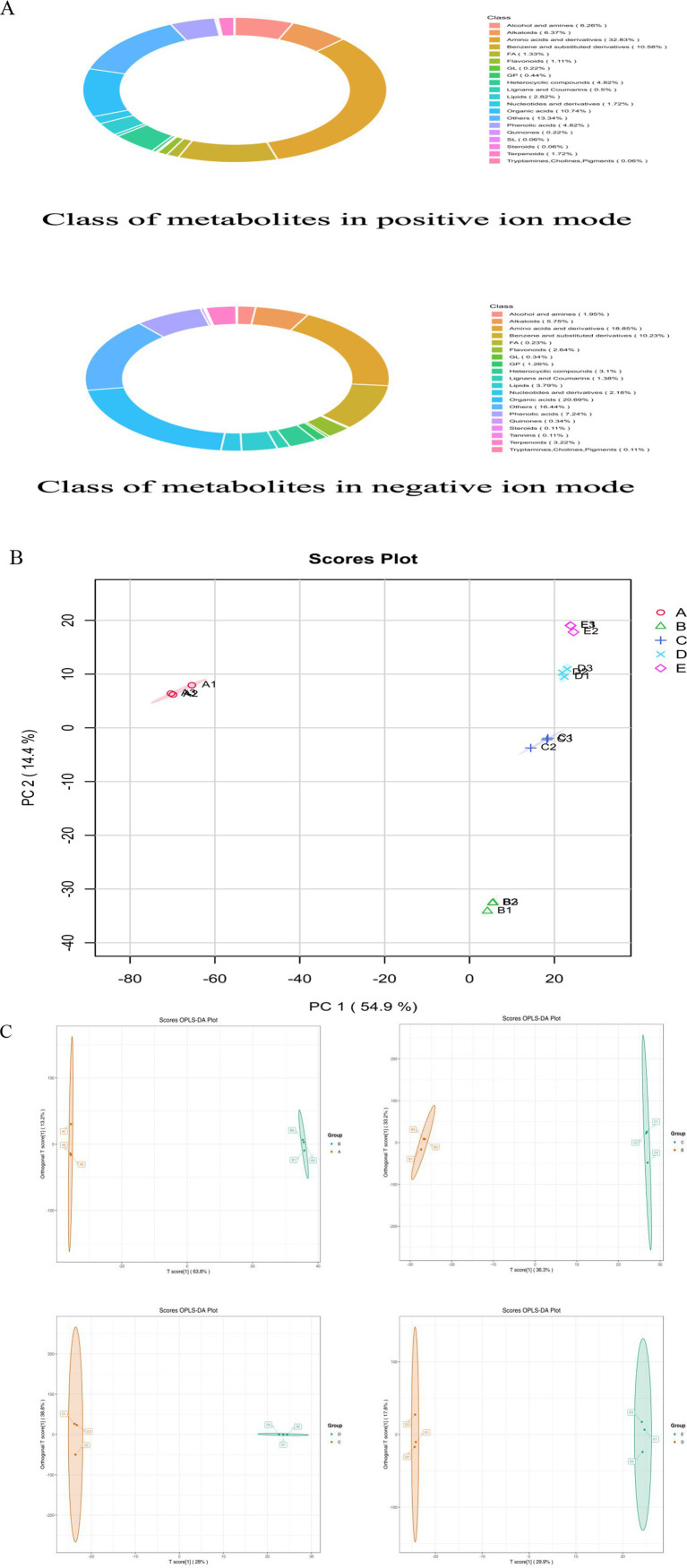
Class of metabolites in positive and negative ion modes **(A)**. Multivariate statistical analysis **(B,C)**. A denoted the non-fermented stage, B denoted the alcoholic-fermentation stage, C denoted the initial acetic acid fermentation stage, D denoted the mid-acetic acid fermentation stage, and E denoted the late acetic acid fermentation stage. Different lowercase letters above each data point indicate significant differences (*P* < 0.05).

To characterize the non-volatile metabolic profile of LVBs across different fermentation stages, an untargeted UHPLC-based metabolomics approach was used to analyze these compounds. Under the detection conditions, the peaks exhibited good symmetry and a relatively uniform distribution. A total of 2,382 metabolites were identified and classified into 20 categories based on the HMDB Taxonomy system at the Class I, with 1,674 and 708 compounds detected in the positive and negative ion modes, respectively (Figure 4A). The most prevalent groups were amino acids and their derivatives, organic acids, benzene and its substituted compounds, alkaloids, alcohols and amines, as well as phenolic acids. A total of 148 alkaloids, 127 alcohols and amines, and 124 phenolic acids were identified. Amino acids and derivatives, organic acids, and benzene and its substituted derivatives were the primary groups, together comprising 52.26% of the total identified metabolites, with roughly 625, 325, and 264 chemicals found, respectively. This suggests that amino acids, organic acids, and benzene, together with their substituted derivatives, jointly constitute the fundamental determinants of the taste quality of LVBs.

Unsupervised PCA was used to assess overall differences in metabolic profiles among samples and within-group variation across distinct fermentation stages (50). In the PCA model, PC1 and PC2 accounted for 46.13 and 13.38% of the total variance, respectively (Figure 4B). The score plot showed clear separation among fermentation stages and distinct clustering within groups. Notably, samples from A were markedly separated from the other four groups, suggesting distinct metabolic profiles across these fermentation stages. In contrast, the D and E groups were positioned close together on the plot, indicating that metabolite composition and relative abundances were similar between the day 35 and day 49 samples.

Given the limited utility of PCA for inter-group discrimination, OPLS-DA, which integrates orthogonal signal correction (OSC) with PLS-DA, was used to improve identification of discriminant metabolites in pairwise comparisons (51). The model was evaluated using three key parameters: *R*^2^*X* and *R*^2^*Y*, representing the explained variance of the *X*- and *Y*-matrices, respectively, and *Q*^2^, indicating predictive capability. Values close to 1 for these parameters indicate a robust and reliable model. A model with *Q*^2^ > 0.5 is generally considered valid, while Q^2^ > 0.9 indicates excellent predictive performance. In this study, OPLS-DA models were established to perform pairwise comparisons between adjacent fermentation groups and to validate metabolite differences in LVBs across fermentation stages. Validation using 200 permutation tests confirmed that models across all groups were robust, with *R*^2^*Y* > 0.794 and *Q*^2^ > 0.534, thereby supporting the interpretation of group differences and subsequent screening of differential metabolites. The OPLS-DA score plot (Figure 4C) demonstrated clear separation of samples across comparative groups, with each group clustering on opposite sides of the confidence interval. This indicates significant alterations in metabolites during LVB fermentation.

#### 3.7.2 Screening and identification of differential metabolites

**Figure 5:**
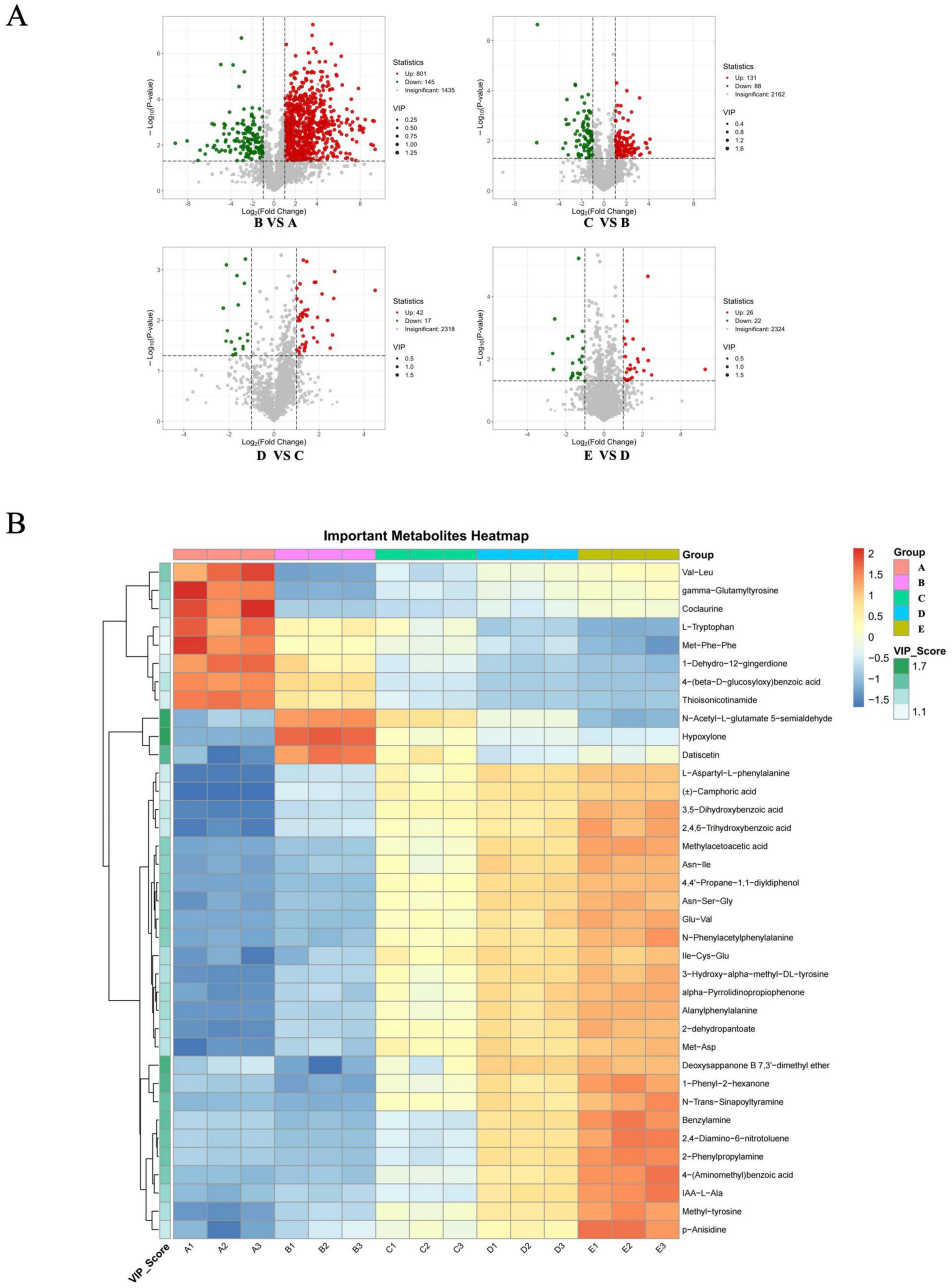
Change of differential metabolites during LVBs fermentation. **(A)** Differential metabolites at different stages during LVB fermentation. **(B)** The heatmap of important differential metabolites. A denoted the non-fermented stage, B denoted the alcoholic-fermentation stage, C denoted the initial acetic acid fermentation stage, D denoted the mid-acetic acid fermentation stage, and E denoted the late acetic acid fermentation stage. Different lowercase letters above each data point indicate significant differences (*P* < 0.05).

During fermentation, substantial changes were observed in metabolite profiles and relative abundances within the LVBs. According to OPLS-DA, 1100 metabolites were identified during fermentation using the criteria VIP > 1.0 and fold change (FC ≥ 2 or ≤0.5) (40). Figure 5A shows that the identification of differential metabolites yielded 946, 219, 59, and 48 distinct material peaks for the comparisons of B vs. A, C vs. B, D vs. C, and E vs. D, respectively. Of these, 801, 131, 42, and 26 peaks exhibited up-regulation, while 145, 88, 17, and 22 peaks showed down-regulation (Figure 5A).

The HMDB databases were used to annotate and identify 37 differential metabolites with a *p*-value threshold of *p* < 0.05 during litchi vinegar-like beverage fermentation (Supplementary Table S4), including amino acids and derivatives (37.8%), organic acids (13.5%), benzene and substituted derivatives (13.5%), ketone compounds (8.1%), alkaloids (5.4%), phenolic acids (2.7%), flavonoids (2.7%), and others. The heatmap (Figure 5B) shows changes in the abundance of differential metabolites across distinct fermentation stages. The hierarchical clustering heatmap of important metabolites (VIP scores ≥ 1.1) showed that all five LVB fermentation stages (A–E) exhibited stage-dependent metabolic profiles, with significant, biologically meaningful clusters. Most notably, non-fermented LVBs (stage A) had significantly higher levels of several dipeptide and aromatic amide derivatives (including Val-Leu, gamma-glutamyltyrosine, coclaurine, *L*-tryptophan, and Met-Phe-Phe) as well as phenolic compounds (such as 1-dehydro-12-gingerdione and 4-(beta-d-glucosyloxy)benzoic acid) than fermented LVBs, indicating that these fruit-derived metabolites are mostly of fruit origin and are gradually consumed or converted during fermentation. After alcoholic fermentation (stage B), there was a general decrease of these fruit-derived metabolites, and a moderate increase of intermediate compounds reflecting active microbial metabolic activity. Transitional metabolic profiles were observed for groups C and D, corresponding to the initial and mid-stage acetic acid fermentation, respectively. Most of these occurred at intermediate-to-high abundance levels, indicating microbial biotransformation. Interestingly, group E (late acetic acid fermentation) showed the greatest dissimilar metabolic fingerprinting compared with the other four fermentation stages, driven by the significant accumulation of a specific cluster of bioactive compounds, i.e., *N*-*trans*-sinapoyltyramine, benzylamine, 2,4-diamino-6-nitrotoluene, 2-phenylpropylamine, 4-(aminomethyl)benzoic acid, IAA-L-Ala, methylytyrosine, and *p*-anisidine. This indicates that the extended acetic acid fermentation promotes *de novo* biosynthesis or release of nitrogenous aromatic metabolites, which may contribute to the unique flavor characteristics and bioactive properties of mature LVBs. Regarding 2,4-diamino-6-nitrotoluene, a suspected carcinogen and mutagen belonging to the benzene and its substitutes, first identified as a nitroaromatic compound derived from TNT, not known to be biosynthetically produced by any member of the fermentation microbiota or in litchi fruit, and structurally similar in mass to several isomers, it was considered a possible false-positive annotation and requires further confirmation using an authentic compound in future studies.

In particular, amino acids and organic acids are key components of vinegar, playing an important role in its taste profile and sensory quality (27). Amino acids, which react with saccharides to form low-molecular-weight flavor compounds and pigments, are the primary metabolites produced by microbial degradation of proteins in raw materials during fermentation (27, 52). This study demonstrates that dynamic changes in free amino acids and dipeptides are key features of amino acid metabolism during LVBs fermentation. These alterations significantly contribute to both the flavor profile and the bioactivity of LVBs (Figure 5B).

The dipeptides Glu-Val (sweet) and Val-Glu (umami and sour), along with the free amino acid *L*-tryptophan, associated with astringency and bitterness (53, 54), showed distinct patterns and functions. The ongoing decrease in L-tryptophan levels correlated with a possible reduction in unpleasant bitter flavors. The ongoing buildup of Glu-Val presumably enhanced sweet perception. The Val level showed a general decline, reaching a low point during the B stage, followed by a persistent rise. Alanylphenylalanine, which is continually increasing, is a bioactive peptide with antihypertensive characteristics (55). Lan et al. reported that Val-Glu and Met-Leu are effective DPP-IV inhibitors, influencing blood glucose regulation by inhibiting this enzyme (56), thereby offering an approach to the development of innovative LVB-based medicines for the treatment of type 2 diabetes. The concentration of 5-methoxytryptophan, an indole metabolite with recognized anti-inflammatory properties (57), increased consistently, whereas its bitter precursor, tryptophan, diminished (Supplementary Figure S3), signifying an inverse correlation in their relative abundances.

Organic acids contribute to the characteristic sourness and stability of fermented foods, influence the formation of ester flavor compounds in fruit vinegar, inhibit the growth of spoilage microorganisms, and exhibit bioactive properties (52). Litchi, rich in reducing sugars, sugar alcohols, and sugar acids, provides an ideal substrate for microbial organic acid production during fermentation (58). Microorganisms hydrolyze fruit proteins and carbohydrates into smaller molecules, which are then converted to pyruvate via pathways such as glycolysis and the hexose monophosphate pathway, ultimately yielding organic acids (59). Additionally, under aerobic conditions, acetic acid bacteria transform ethanol and sugar alcohols into organic acids. The tricarboxylic acid (TCA) cycle also generates various organic acids during the metabolism of carbohydrates, lipids, and amino acids (52). After fruit vinegar fermentation, the relative abundances of 3,5-dihydroxybenzoic acid, (±)-camphoric acid, 4-(aminomethyl) benzoic acid, and 2-dehydropantoate increased. In contrast, 4-(beta-*D*-glucosyloxy) benzoic acid decreased throughout the process.

4-(beta-*D*-Glucosyloxy) benzoic acid (also known as 4-hydroxybenzoic acid 4-O-glucoside), a major conjugated form of 4-hydroxybenzoic acid in plant cell walls, is a plant-derived phenolic acid (58). During fermentation, microorganisms’ enzymatic activities cause enzymolysis and disruption of the cell wall and membrane, thereby significantly increasing the release and free-form content of these active metabolites in fermented fruits. Han et al. (60) demonstrated that fermenting Lychee with mixed probiotic strains promoted the release of 4-hydroxybenzoic acid 4-*O*-glucoside. Contrary to our experimental observations, the continuous decrease in 4-hydroxybenzoic acid 4-*O*-glucoside during LVB fermentation might be attributed to enzymatic deglycosylation, with the resulting 4-hydroxybenzoic acid (4-HBA) subsequently increasing (Supplementary Figure S4).

### 3.8 Correlation of microbial community with non-volatile flavor metabolites during spontaneous fermentation

**Figure 6:**
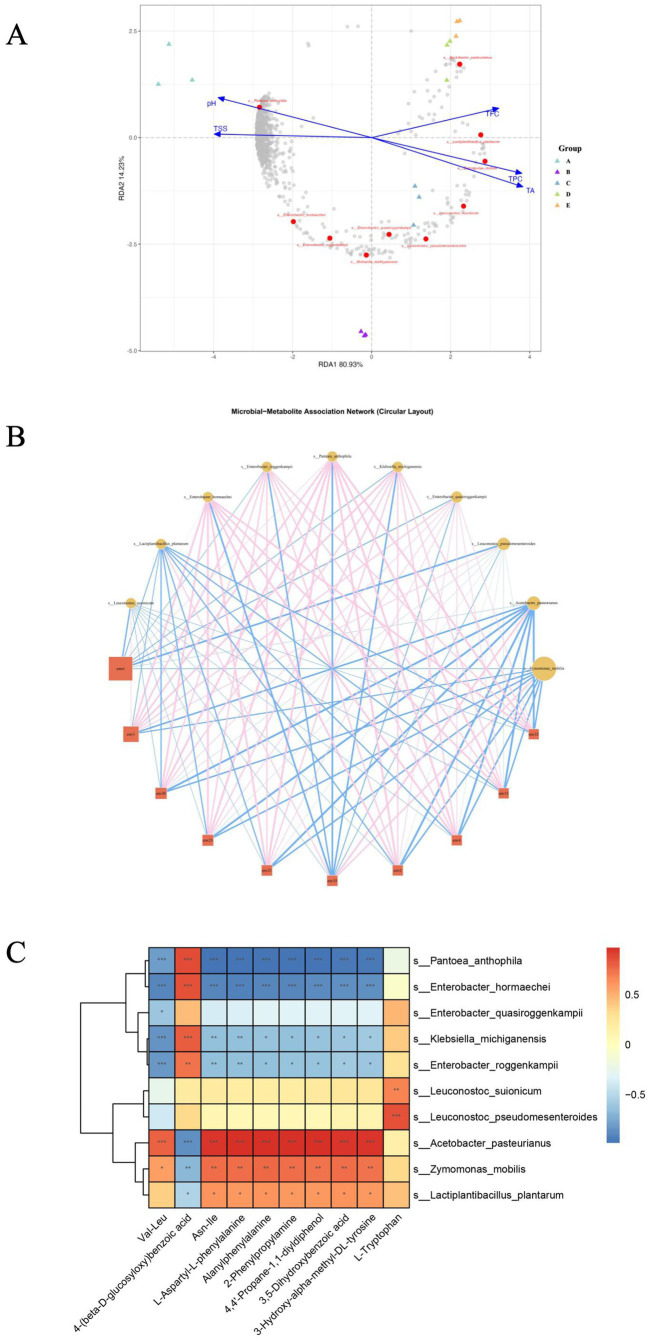
The correlation between microbial community and physicochemical parameters and non-volatile flavor metabolites in LVBs fermentation. **(A)** RDA of the dominant microbes and physicochemical parameters. The network **(B)** and heatmap **(C)** between the dominant microbes and key non-volatile metabolites during LVB’s spontaneous fermentation. A denoted the non-fermented stage, B denoted the alcoholic-fermentation stage, C denoted the initial acetic acid fermentation stage, D denoted the mid-acetic acid fermentation stage, and E denoted the late acetic acid fermentation stage. Different lowercase letters above each data point indicate significant differences (*P* < 0.05).

During vinegar fermentation, microbial metabolic activity drives dynamic shifts in the environment, imposing selective pressure that leads to the self-domestication of the microbial community and a directed succession toward a more fermentation-adapted structure, ultimately resulting in orderly changes in its function and interspecific interactions (61). In this study, redundancy analysis (RDA) was performed using the vegan package in R to further elucidate correlations between dominant microbes and physicochemical factors during LVB fermentation. RDA1 and RDA2 accounted for 80.93 and 14.23% of the variation in microbial community structure, respectively, totaling 95.16% (Figure 6A), indicating that the selected environmental factors have strong explanatory power for LVB microbial community structure. Triangles of different colors represent different samples. The dots represent microbial communities, with red dots indicating the top 10 most abundant characteristic communities and gray dots representing the remaining communities. In the RDA, the closer a sample is to a species, the higher that species’ relative abundance in the sample (62). As shown in Figure 6A, microbial communities were predominantly distributed in the second quadrant, followed by the third and fourth quadrants, with the fewest observed in the first quadrant. This indicates that the number of species in groups D and E was significantly lower than in groups A, B, and C, further confirming that the microbial community gradually declined during fermentation.

The RDA identified TSS as the strongest independent driver of microbial community variation (*R*^2^ = 0.768, *F* = 47.37, *p* = 0.002), followed by TA (*R*^2^ = 0.821, *F* = 4.80, *p* = 0.020). Marginal effect analysis indicated that pH also had a substantial effect (*F* = 59.38), likely due to its collinearity with TSS. In contrast, TPC and TFC showed minimal effects (*F* = 1.59 and 1.26, respectively), with TFC having the weakest influence. These results suggest that TSS and TA were the primary environmental drivers, while TFC had the smallest impact on LVB community structure.”

Among them, TA showed a strong positive correlation with *Zymomonas mobilis* and *Leuconostoc suionicum*. *Pantoea anthophila* was positively correlated with pH and negatively correlated with TA, indicating that these microorganisms are particularly sensitive to acidic conditions and may regulate microbial competition through acidity, thereby driving changes in their populations and abundance. As fermentation progresses, the dominant microbial communities undergo continuous succession, enabling them to adapt to changing environmental conditions and sustain their metabolic activities. This dynamic adaptation ensures the survival of these microbial communities and preserves their essential role in producing flavor compounds.

Microbial metabolites are the primary contributors to the vinegar-like beverage’s unique flavor and function. To investigate associations between microorganisms and metabolites in the fermentation system, network plots (Figure 6B) and heatmaps (Figure 6C) were used to display Spearman correlations between the dominant microbial communities and the 37 characteristic differential metabolites. The correlation network revealed a highly structured metabolic interaction network between microorganisms and metabolites (Figure 6B). Within the positively correlated cluster, *Zymomonas mobilis* and *Acetobacter pasteurianus* played prominent roles, showing significant correlations with Val-Leu (env1), 2-phenylpropylamine (env21), 4,4′-propane-1,1-diyldiphenol (env33), alanylphenylalanine (env2), *L*-aspartyl-*L*-phenylalanine (env4), Asn-Ile (env23), 3-hydroxy-alpha-methyl-*DL*-tyrosine (env13), and 3,5-dihydroxybenzoic acid (env30). Additionally, *Lactiplantibacillus plantarum* showed positive correlations with *Acetobacter pasteurianus*. Notably, *Z. mobilis* showed strong positive correlations with ethanol-related metabolites, consistent with its known ethanologenic potential; *A. pasteurianus* exhibited positive associations with acetic acid and negative associations with ethanol, consistent with its known role in acetic acid oxidation; and *Lactiplantibacillus plantarum* showed positive correlations with organic acids, suggesting a potential role in acid accumulation. In contrast, a negatively correlated cluster, represented by Pantoea and *Enterobacter roggenkampii*, showed significant negative correlations with these metabolites, potentially reflecting niche differentiation or resource competition within the microbial community. It should be noted that these correlations reflect covariation patterns rather than causal relationships; the specific metabolic contributions of individual taxa require further functional validation.

## 4 Conclusion

In summary, the spontaneous fermentation of litchi vinegar-like beverages (LVBs) exhibited highly dynamic ecological structuring of microbial succession, with community diversity gradually decreasing as functional specialization increased across successive fermentation stages. Additionally, the dominant ethanol-producing organism was identified as *Zymomonas mobilis*, which serves as a substrate for the subsequent oxidation by *Acetobacter*. This distinguishes LVB fermentation from traditional cereal-based vinegar fermentation systems in which *Saccharomyces cerevisiae* dominates the alcoholic fermentation stage. Additionally, correlation network analysis showed that the two physico-chemical parameters, titratable acidity, and total soluble solids were found to be the main environmental factors influencing the structural organization of microbial communities and the composition of metabolic products. The metabolic interactions among the core fermentative species (*Zymomonas*, *Acetobacter*, *Leuconostoc*, and *Lactiplantibacillus*) and competition for niches by *Enterobacter* regulated the synthesis and transformation of flavor- and bioactivity-related metabolites, including tryptophan, 3,5-dihydroxybenzoic acid, and 4-(*β*-*D*-glucosyl)-*O*-benzoic acid.

It should be noted that the inherent variability of spontaneous fermentation precludes direct extrapolation to standardized production; rather, the value of this study lies in revealing the microbial–metabolite association patterns that may guide future rational design of starter cultures and fermentation strategies. In addition, the sampling design was suitable for the capture of the most important physiological changes of the LVB fermentation, but only two timepoints were sampled during the alcoholic fermentation stage (days 0 and 7). Further research with more frequent sampling (e.g., daily samples) during this early period (as the microbial succession is very dynamic and swift) would generate more granular detail in the underlying microbial succession.

## Data Availability

The original contributions presented in the study are included in the article/Supplementary material, further inquiries can be directed to the corresponding authors.

## Footnotes

1
https://www.bioincloud.tech
