# Supplementary material for: Multi-omics profiling of microbial ecology and non-volatile compounds across fermentation stages of spontaneous litchi (Litchi chinensis Sonn.) fermented vinegar-like beverage
Source: Front Nutr. 2026 Jul 30;13:1908193. doi: 10.3389/fnut.2026.1908193 (PMC13467820; doi:10.3389/fnut.2026.1908193)
Supplement: Supplementary file 1 [file Image_1.pdf]

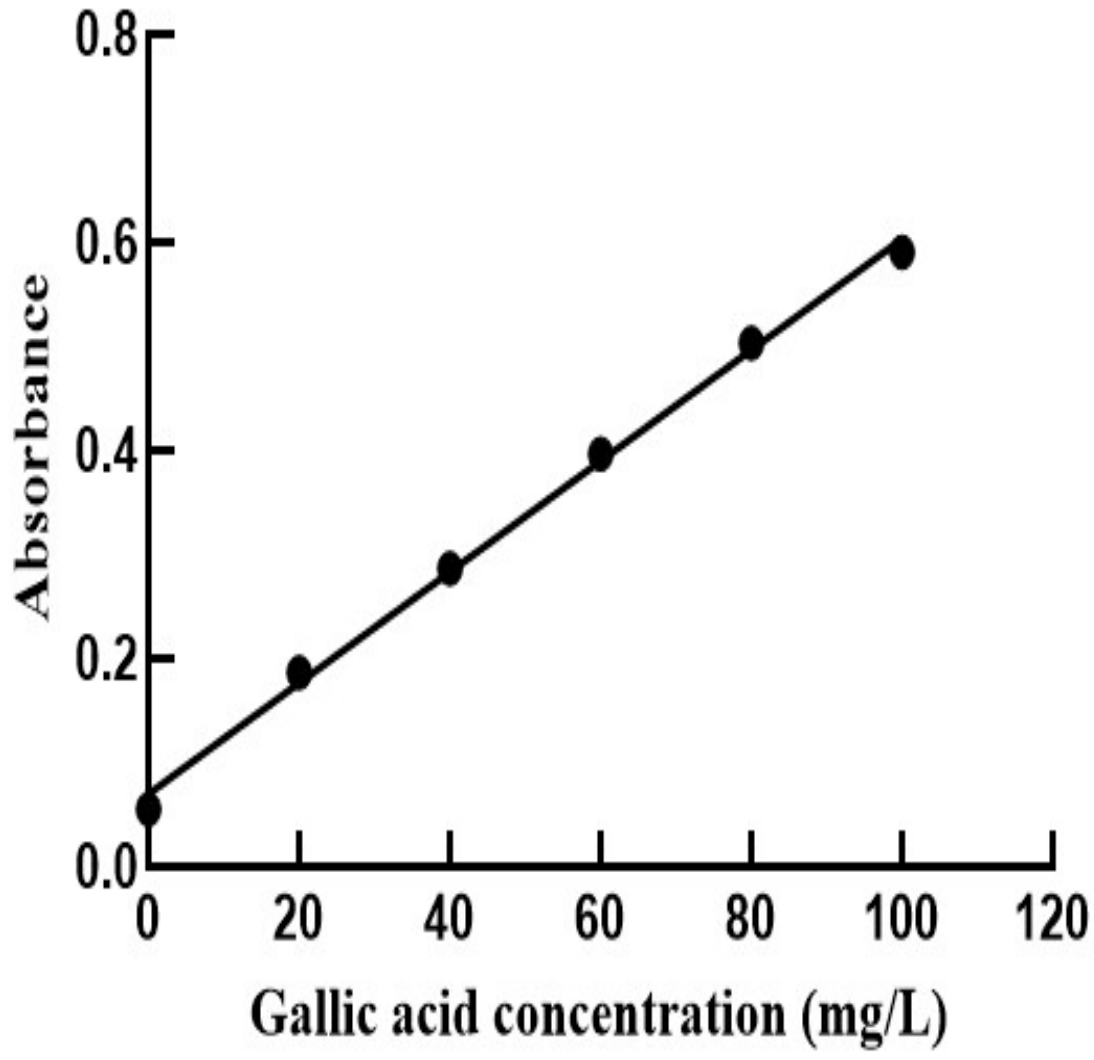

(A)

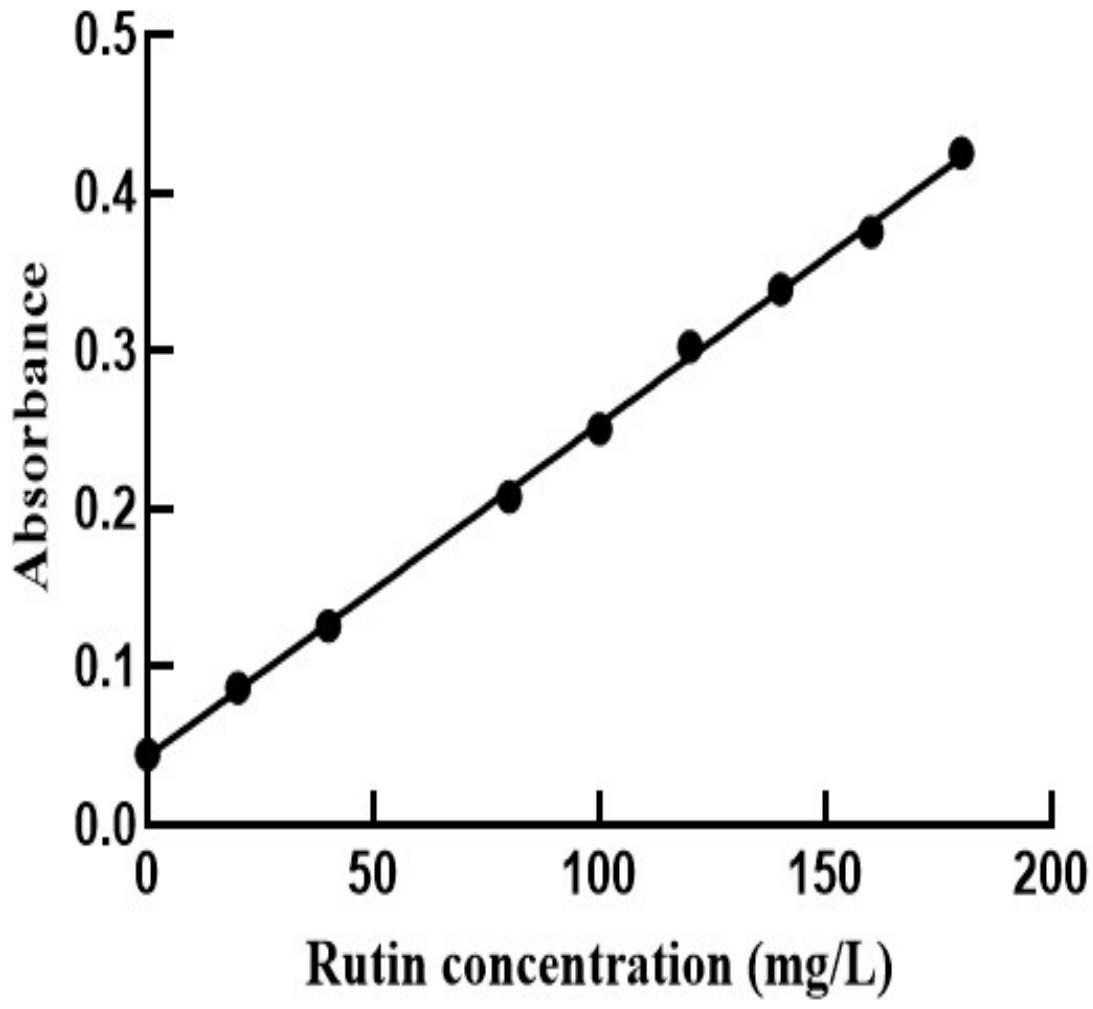

(B)

FIGURE S1. Standard curves for gallic acid equivalent (A) and rutin (B). (A) Gallic acid equivalent (GAE) standard curve: A linear relationship was observed between absorbance (Y) and gallic acid concentration (X), yielding the equation Y = 0.005334*X + 0.07006 (R^2 = 0.9972) in the range of 0–100 mg/L. (B) Rutin equivalent standard curve: A linear relationship was observed between absorbance (Y) and rutin concentration (X), yielding the equation Y = 0.002106*X + 0.04333 (R^2 = 0.9992) in the range of 0–180 mg/L.

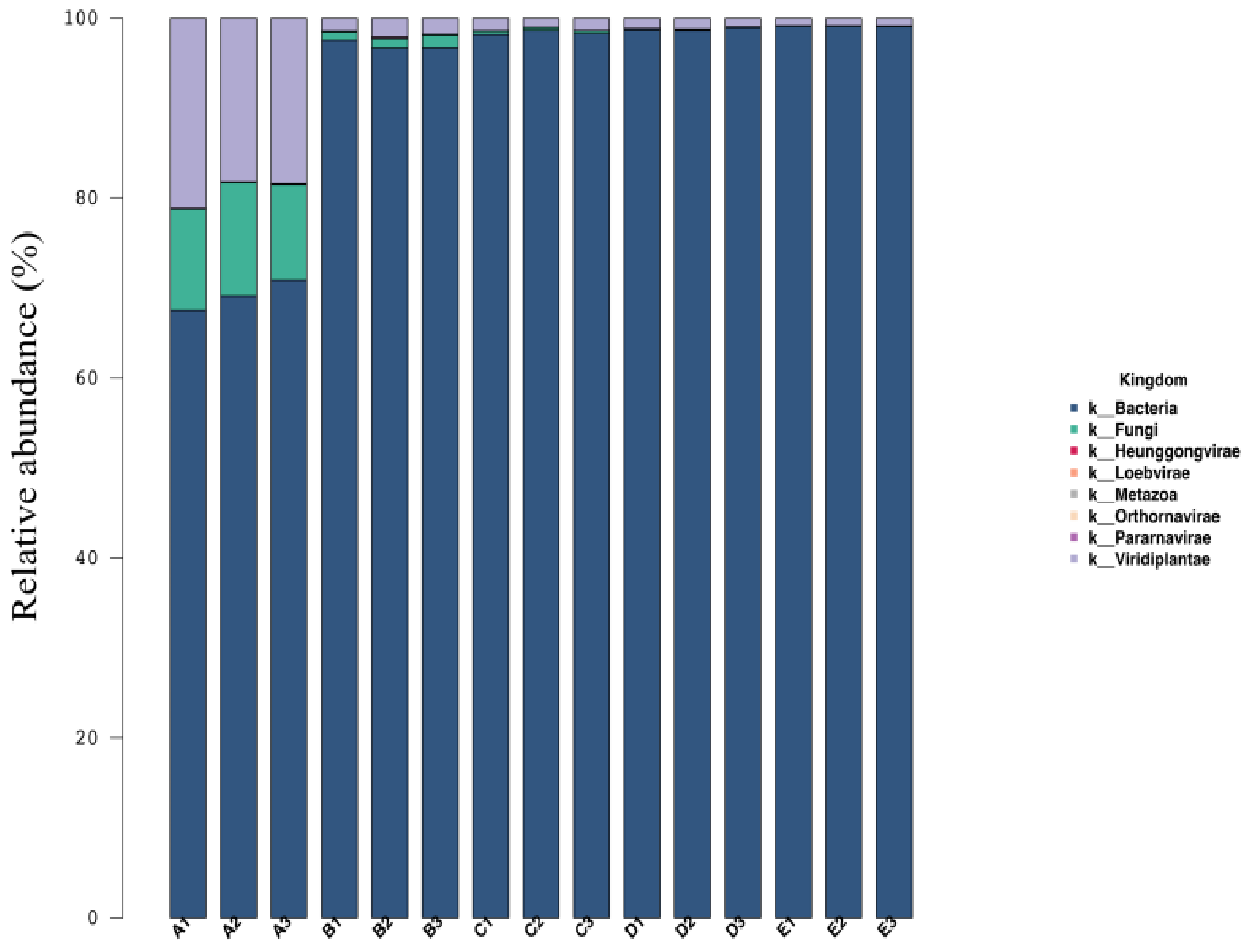

FIGURE S2. The pattern of community succession at the kingdom level during LVB spontaneous fermentation.

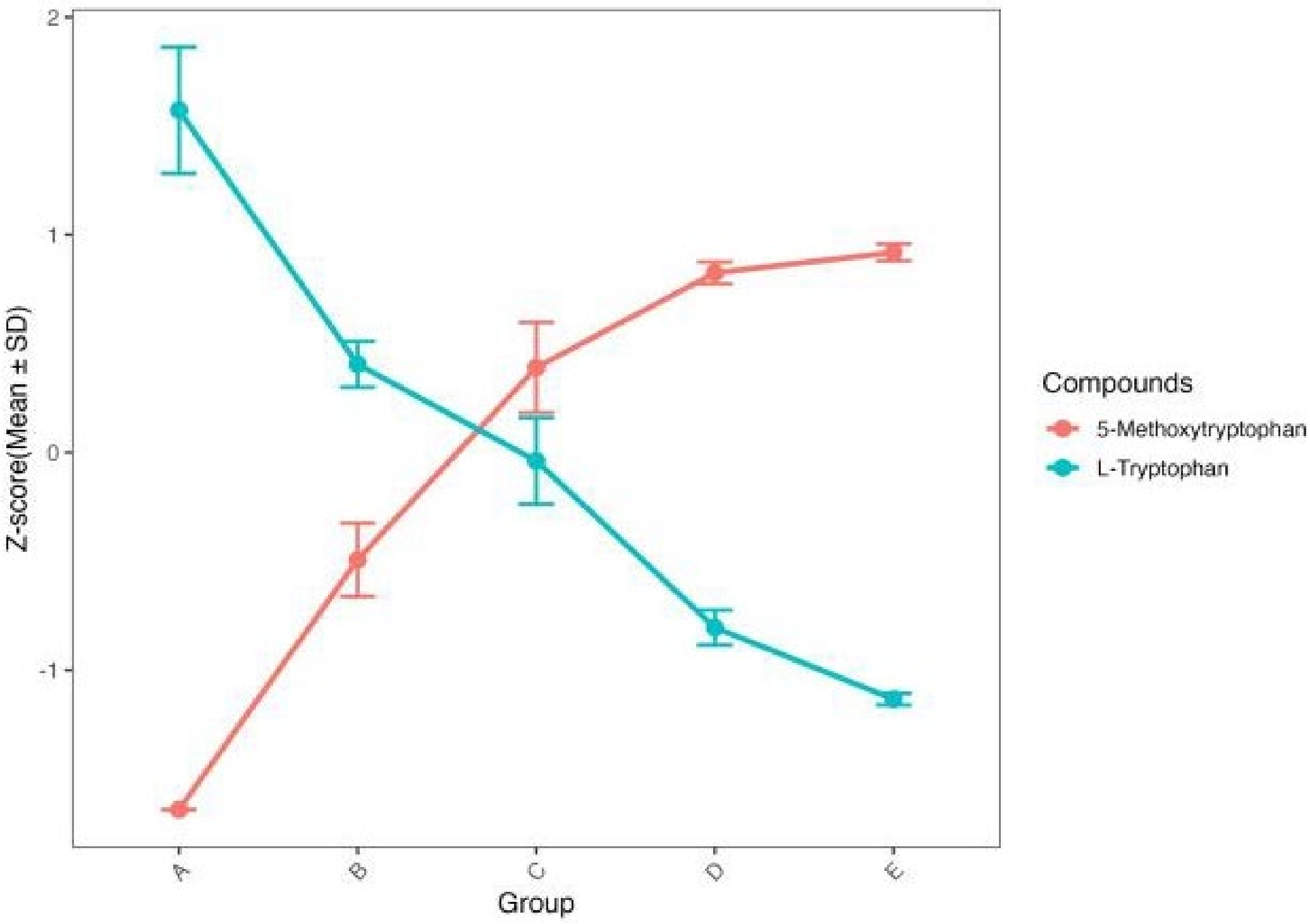

FIGURE S3. Change trends of 5-methoxytryptophan and *L*-tryptophan across different spontaneous fermentation stages of LVB.

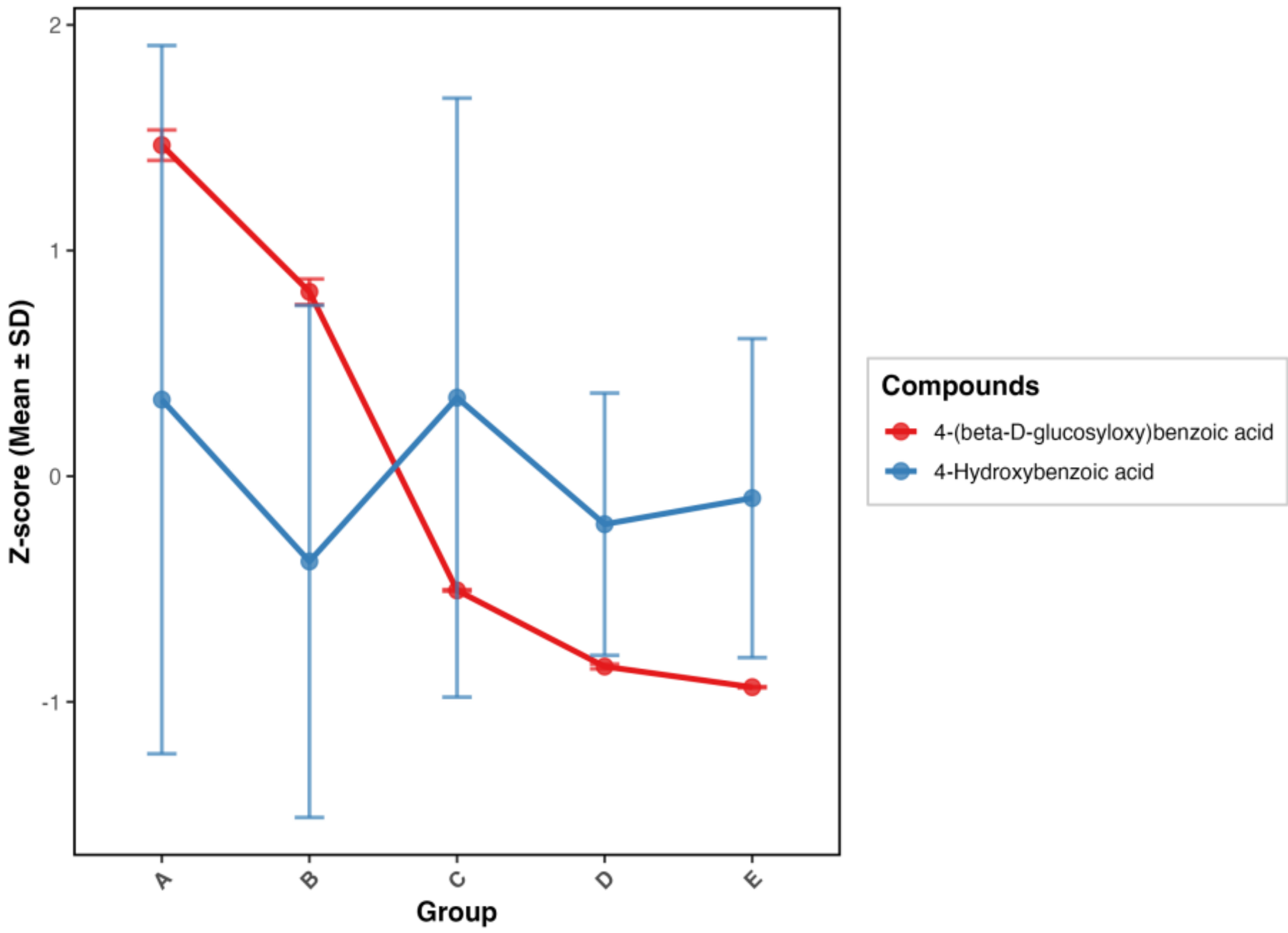

FIGURE S4. Change trends of 4-(beta-*D*-glucosyloxy) benzoic acid and 4-hydroxybenzoic acid across different spontaneous fermentation stages of LVB.
